# Activation of Endocannabinoid System Is Associated with Persistent Inflammation in Human Aortic Aneurysm

**DOI:** 10.1155/2015/456582

**Published:** 2015-10-11

**Authors:** Christopher Gestrich, Georg D. Duerr, Jan C. Heinemann, Anne Meertz, Chris Probst, Wilhelm Roell, Wolfgang Schiller, Andreas Zimmer, Laura Bindila, Beat Lutz, Armin Welz, Oliver Dewald

**Affiliations:** ^1^Department of Cardiac Surgery, University Clinical Centre Bonn, Sigmund-Freud Street 25, 53105 Bonn, Germany; ^2^Institute of Molecular Psychiatry, Life & Brain Center, Sigmund-Freud Street 25, 53105 Bonn, Germany; ^3^Institute of Physiological Chemistry, University Medical Centre of the Johannes Gutenberg University Mainz, Duesbergweg 6, 55128 Mainz, Germany

## Abstract

Human aortic aneurysms have been associated with inflammation and vascular remodeling. Since the endocannabinoid system modulates inflammation and tissue remodeling, we investigated its components in human aortic aneurysms. We obtained anterior aortic wall samples from patients undergoing elective surgery for aortic aneurysm or coronary artery disease as controls. Histological and molecular analysis (RT-qPCR) was performed, and endocannabinoid concentration was determined using LC-MRM. Patient characteristics were comparable between the groups except for a higher incidence of arterial hypertension and diabetes in the control group. mRNA level of cannabinoid receptors was significantly higher in aneurysms than in controls. Concentration of the endocannabinoid 2-arachidonoylglycerol was significantly higher, while the second endocannabinoid anandamide and its metabolite arachidonic acid and palmitoylethanolamide were significantly lower in aneurysms. Histology revealed persistent infiltration of newly recruited leukocytes and significantly higher mononuclear cell density in adventitia of the aneurysms. Proinflammatory environment in aneurysms was shown by significant upregulation of M-CSF and PPAR*γ* but associated with downregulation of chemokines. We found comparable collagen-stained area between the groups, significantly decreased mRNA level of CTGF, osteopontin-1, and MMP-2, and increased TIMP-4 expression in aneurysms. Our data provides evidence for endocannabinoid system activation in human aortic aneurysms, associated with persistent low-level inflammation and vascular remodeling.

## 1. Introduction

Aneurysms of the thoracic aorta include a wide range of genetic, degenerative, and acquired disease conditions and may result in a life-threatening Stanford type A dissection, needing emergency cardiac surgery [1]. Conservative therapy options are limited during the development of aortic aneurysm, and therefore surgical replacement of the ascending aorta or endovascular therapy for descending aorta is the treatment of choice [2]. In the last few years, an increased number of clinical and experimental studies contributed to a better understanding of the mechanisms involved in the development of aortic aneurysms. Genetic diseases such as Marfan syndrome, Ehlers-Danlos syndrome, and Loeys-Dietz syndrome are well described, but relatively rare [3, 4]. A combination of causes is assumed in patients with bicuspid aortic valve, which have a higher risk of aortic dilation or dissection, probably due to altered regional hemodynamics and structural anatomy of the aortic wall [5, 6]. Inflammation has also been suggested in the development of abdominal aortic aneurysms, since cellular infiltration via vasa vasorum was observed in the aortic wall and associated with expression of inflammatory mediators [7, 8]. Because of differences in aetiologies [9], these findings from abdominal aneurysms cannot be directly projected into thoracic aneurysms, where we lack an evidence for inflammation. Nevertheless, involvement of mediators of vascular remodeling, for example, metalloproteinases 2 and 9 (MMP-2 and MMP-9), as well as oxidative stress has also been shown in studies investigating aortic aneurysm specimen [10–12].

The role of the endocannabinoid system in homeostasis and pathology has been established in most of the organs and body systems [13]. Cannabinoid CB2 receptor and its ligands anandamide and 2-arachidonoyl-glycerol have been associated with the regulation of inflammatory response in several conditions [14]. An experimental study postulated an antifibrotic role for the CB2 receptor in a model of liver fibrosis [15]. Also, the endocannabinoid anandamide has been associated with the regulation of pulmonary vascular resistance [16]. Furthermore, the CB2 receptor acts in cardioprotective manner and influences myocardial remodeling in a murine model of ischemic cardiomyopathy [17]. Our recent work showed the activation of the endocannabinoid system and its association with persistent inflammatory reaction in human myocardial hypertrophy of patients with aortic stenosis [18].

We therefore investigated cannabinoid receptors and their ligands and their association with persistent inflammation and vascular remodeling in human aortic aneurysms.

## 2. Materials and Methods

### 2.1. Patient Data

The ethics committee of the Medical School at the University of Bonn approved the study protocol and the investigation is conformed to the principles outlined in the Declaration of Helsinki. All patients gave an informed consent to participate in this study. Samples of the anterior aortic wall were collected from patients undergoing elective surgery for aortic aneurysm, defined as diameter of ascending aorta >5.0 cm (*n* = 19) or for coronary artery disease as controls (*n* = 73). Small buttons of aortic tissue from coronary artery bypass grafting (CABG) patients were collected from a single patient for histological evaluation (*n* = 24) or pooled for molecular analysis (*n* = 30) and mass spectrometry (*n* = 25), because of the small tissue amount available from one button. In order to exclude possible differences between the control patients for each specific analysis, we performed statistical analysis not only between total CABG and aneurysms, but also between the CABG subgroups, as well as every subgroup versus aneurysms (Table 1). Patients with a positive family history, chronic dissection, penetrating aortic ulcer, Marfan syndrome, or other genetic disorders, as well as tumour disease, were excluded.

The analysis of control subgroups showed no significant differences in any parameter between the three subgroups. Patients in the total control group had significantly lower incidence in aortic regurgitation and bicuspid aortic valve than in aneurysms (Table 1). Also, patients in total control group had significantly higher incidence of arterial hypertension and diabetes mellitus, as well as higher white blood cell count, despite being in lower normal range. Based on surgery reports, we found mild to moderate signs of atherosclerosis with calcifications in 57.80% (11/19) of aneurysms, while palpable aortic calcifications were reported in 61.64% (45/73) of patients in the control group. All other parameters were comparable between the total controls and aneurysms. For significant differences between each control subgroup and aneurysms, please refer to Table 1. The perioperative data is summarized in Table 2. The control group patients underwent either solitary CABG or combined CABG and aortic valve replacement. The patients with aortic aneurysm underwent replacement of the ascending aorta with either aortic valve repair using partial excision of the noncoronary part of the aortic bulbus or aortic valve replacement using a bioprosthesis. The postoperative stroke rate and laboratory parameters except for troponin were not significantly different between the groups.

### 2.2. Endocannabinoid Quantification by Liquid Chromatography-Multiple Reaction Monitoring

#### 2.2.1. Chemicals and Standard Solutions

Anandamide (AEA), 2-arachidonoyl glycerol (2-AG), arachidonic acid (AA), and their deuterated analogues AEA-d4, 2-AG-d5, and AA-d8 were obtained from Cayman Chemicals (Ann Arbor, MI, USA). Water, acetonitrile (ACN), formic acid (FA), ethyl acetate, and hexane (all from Fluka LC-MS grade) were obtained from Sigma-Aldrich.

#### 2.2.2. Endocannabinoid Extraction

For eCBs extraction, heart tissues were first weighted in the cold room and transferred to precooled 2 mL Precellys tubes containing cold ceramic beads. Spiking solution of deuterated eCBs in acetonitrile (50 *μ*L) was mixed with 450 *μ*L ethyl acetate/hexane (9 : 1, v/v) and added to the tissue samples, followed by 0.1 M FA (e.g., 600 *μ*L of 0.1 M FA and 400 *μ*L of 0.1 M FA were added for aorta tissue and control tissue, resp.). Samples were homogenized for 2 min at 5000 rpm with Precellys 24 (Bertin Technologies, Montigny-le-Bretonneux, France). Homogenates were then centrifuged at 10000 g and 4°C for 10 min then at 16000 g for another 10 min and then kept for 10 min at −20°C to freeze the aqueous phase. The upper organic phase was recovered and evaporated to dryness and the extracts were reconstituted in 50 *μ*L water : acetonitrile (1 : 1, v/v) for further LC/MRM analysis. Throughout the extraction procedure, the tubes, plates, beads, and so forth were invariably precooled and kept at 4°C. The samples were as well invariably kept on ice throughout the entire extraction procedure to prevent artificial alterations of endogenous eCB levels originating from enzymatic or chemical degradation and/or* ex vivo* synthesis of eCBs. The amounts of internal standards and concentration range of calibration curves were selected using test heart tissues.

#### 2.2.3. Equipment

Liquid chromatography-multiple reaction monitoring (LC-MRM) analyses were performed on a LC-MS/MS system consisting of a 5500 QTrap triple-quadrupole linear ion trap mass spectrometer equipped with a Turbo V Ion Source (AB SCIEX, Darmstadt, Germany), an Agilent 1200 series LC system (degasser, pump; Agilent, Waldbronn, Germany), and a CTC HTC PAL autosampler (CTC Analytics AG, Zwingen, Switzerland). Data acquisition and analysis were performed using Analyst software (version 1.6.1; AB SCIEX).

#### 2.2.4. LC-MRM Quantification

20 *μ*L of the solution of extracted eCBs was injected and separated on a Phenomenex Luna 2.5 *μ*m C18(2)-HST column, 100 mm × 2 mm, combined with a precolumn (C18, 4 mm × 2 mm; Phenomenex, Aschaffenburg, Germany), by increasing acetonitrile containing 0.1% formic acid over 2 min from 55% to 90% and maintaining it at 90% for 5.5 min. Throughout the analysis, samples were maintained at 4°C in the LC autosampler. The separated eCBs were flow-through analyzed using MRM. Positive ions (of AEA, 2-AG, and PEA) and negative ions (of AA) were simultaneously analyzed using the “positive-negative-switching” mode. The following MRM transitions were monitored for quantification of eCBs: AEA, *m*/*z* 348.3 to *m*/*z* 62.3; AEA-d4, *m*/*z* 352.3 to *m*/*z* 62.1; 2-AG, *m*/*z* 379.1 to *m*/*z* 287.2; 2-AG-d5, *m*/*z* 384.1 to *m*/*z* 287.2; PEA, *m*/*z* 300.2 to *m*/*z* 62.1; PEA-d4, *m*/*z* 304.2 to *m*/*z* 62.1; AA, *m*/*z* 303.05 to *m*/*z* 259.1; and AA-d8, *m*/*z* 311.04 to *m*/*z* 267.0. Endocannabinoid concentrations were normalized to the tissue weight.

### 2.3. Histology and Immunohistochemistry

Basic histological evaluation of paraffin-embedded 5 *μ*m slices was performed using hematoxylin-eosin staining. Collagen area was calculated on picrosirius red stained samples as previously described [17]. Briefly, photographic images were taken (DP70 camera, Olympus, Münster, Germany) and planimetric analysis of collagen-stained area as percentage of the total aortic wall area was performed using the Software Cell-F (Olympus Soft Imaging Solutions, Münster, Germany).

In order to investigate specific cell types, we used the following primary antibodies cross-reacting with human tissue for immunohistochemistry: MAC-387 monoclonal mouse anti-human antibody for newly recruited leukocytes (NatuTec, Frankfurt, Germany) and CD11b monoclonal rabbit anti-human antibody for monocytes (clone: EP1345Y (ab52478); Abcam, Cambridge, UK). An appropriate Vectastain Elite ABC kit and diaminobenzidine (both from AXXORA, Lörrach, Germany) were used for immunohistochemistry. Cell density was calculated using cell count/mm^2^.

### 2.4. Molecular Analysis

mRNA of the aortic wall samples was isolated using standard phenol/chloroform extraction (TRIzol, Invitrogen, Karlsruhe, D). First-strand cDNA was synthesized using the high capacity cDNA transcription kit (Applied Biosystems, Foster City, CA, USA) with random hexameric primers as described by the manufacturer protocol. Expression of mRNA was analysed using the TaqMan real time quantitative PCR system (RT-qPCR; Applied Biosystems). RT-qPCR was performed and analysed on an ABI Prism 7900HT Sequence Detection System and SDS2.2 Software (Applied Biosystems) with 1/10 diluted cDNA following manufacturer's instructions. Target gene-expression was normalized to an internal control and housekeeping gene GAPDH using comparative ΔΔCT method. All primers were measured using FAMTAMRA chemistry and relative standard curve method. Dissociation curve analysis was performed in order to ascertain the amplification.

### 2.5. Statistical Analysis

Sample size calculations were performed using G^*∗*^Power (V3.1) and depending on the expected difference of the given parameter resulted in a minimum of 15 patients needed for each group. The poststatistical calculation confirmed this value. Data are reported as mean ± SEM and tested for normal distribution using D'Agostino and Pearson omnibus normality test. Differences were analysed in Prism 6.0 software (GraphPad, La Jolla, CA, USA) using Mann-Whitney test or unpaired *t*-test and considered statistically significant when *p* < 0.05.

## 3. Results

### 3.1. Activated Endocannabinoid System in Human Aortic Aneurysms

**Figure 1:**
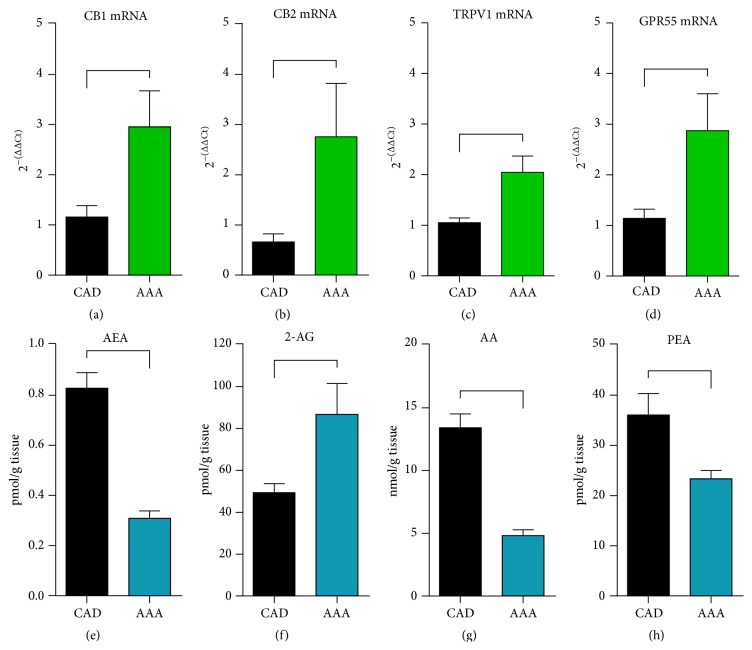
Activation of the endocannabinoid system in aortic aneurysms. mRNA levels of cannabinoid receptors (a) CB1, (b) CB2, (c) TRPV1, and (d) GPR55 showed a significant increase in aneurysm of ascending aorta (AAA) as compared to the normal aorta of patients with coronary artery disease (CAD). Tissue concentration of the endocannabinoids (c) anandamide (AEA) and (d) 2-arachidonoyl glycerol (2-AG), as well as their degradation products (e) arachidonic acid (AA), and (f) palmitoylethanolamide (PEA) in comparison between the groups. *n* = 19 in the AAA group, *n* = 30 in the CAD RT-qPCR, and *n* = 25 in the CAD LC-MRM group. mRNA levels in RT-qPCR are related to controls and GAPDH using comparative ΔΔCt method. Bracket indicates *p* < 0.05.

Experimental data suggested dynamic regulation of endocannabinoids and their receptors in the vascular system [16]. Therefore, we investigated the components of the endocannabinoid system in human aorta and found significantly higher mRNA levels of the cannabinoid receptors CB1 (Figure 1(a)), CB2 (Figure 1(b)), TRPV1 (Figure 1(c)), and GRP55 (Figure 1(d)) in the aneurysms as compared to the samples from controls. Expression of related factors 5HT1A and PPAR*α* was comparable between the groups (data not shown). Mass spectrometry measurements of endocannabinoids showed a significantly lower level of anandamide (Figure 1(c)) and a significantly higher level of 2-arachidonoyl glycerol in aneurysms (Figure 1(d)). Interestingly, aneurysm samples contained a significantly lower amount of the endocannabinoid degradation product arachidonic acid (Figure 1(e)) and palmitoylethanolamide (Figure 1(f)) than the control samples. Therefore, aortic aneurysm showed not only increased level of cannabinoid receptors, but also a different amount of ligands and decreased level of their degradation products suggesting differentiated, persistent action of endocannabinoids in the aortic wall.

### 3.2. Cellular Infiltration of the Aortic Wall and Associated Mediators of Inflammation

**Figure 2:**
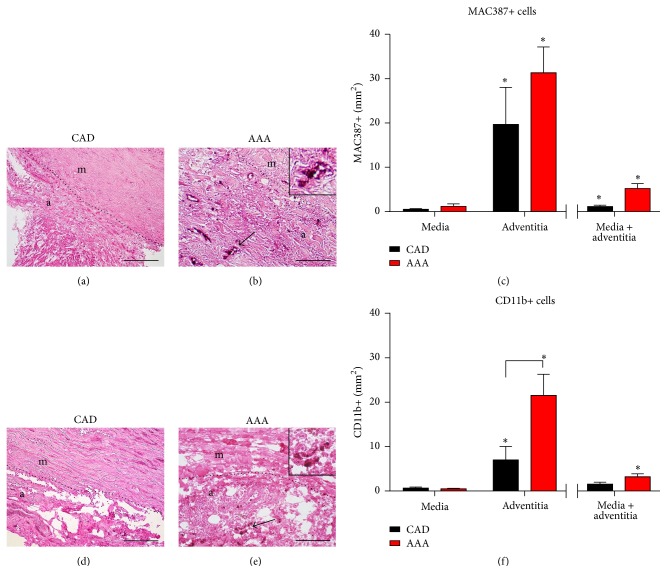
Infiltration of aortic wall with inflammatory cells. Representative image of newly recruited leukocytes stained with MAC-387 antibody (a) in aorta from patients with coronary artery disease (CAD) and (b) from aneurysm of the ascending aorta (AAA). Arrows indicate the stained leukocytes and the dotted line delineates the adventitia (a) from the media (m). (c) Quantitative analysis of newly recruited leukocytes showed comparable cell density between the groups with significantly higher density in adventitia than in the media. Representative image of mononuclear cells stained using CD11b antibody in (d) aorta from CAD and (e) from AAA patient. (f) Quantitative analysis of mononuclear cells showed significantly higher cell density in adventitia of AAA samples than in CAD samples or respective media of the aortic wall. Scale bar in (a), (b), (d), and (e): 200 *μ*m. *n* = 19 in the AAA group and *n* = 24 in the CAD histology group. Bracket indicates *p* < 0.05 between the groups; *∗* indicates *p* < 0.05 versus media.

Animal studies and our previous work in human myocardial hypertrophy showed association between activation of endocannabinoid system and persistent inflammatory reaction [14, 18]. Therefore, we investigated the infiltration of the aortic wall with inflammatory cells. We quantified newly recruited leukocytes stained with MAC387 and found a significantly higher cell density of them in the adventitia of both groups when compared to the respective tunica media (Figures 2(a)–2(c)). We observed a comparable density of newly recruited leukocytes between the aneurysms and controls in the adventitia, indicating persistent accumulation of inflammatory cells into the aortic wall in both groups. Since mononuclear cells differentiate into macrophages after tissue invasion and macrophages act as an important source of different inflammatory or tissue remodeling mediators, we evaluated their infiltration into aortic wall using CD11b staining. Mononuclear cell density was significantly higher in adventitia of aneurysms not only when compared to controls, but also when compared to the media (Figures 2(d)–2(f)). At the same time, control samples showed no significant difference in mononuclear cell density between complete wall and adventitia, but significantly more cells in the adventitia alone. Taken together, the aneurysms and controls showed persistent infiltration of aortic wall with newly recruited leukocytes, but the aneurysms presented with a significantly higher density of tissue remodeling relevant mononuclear cells.

**Figure 3:**
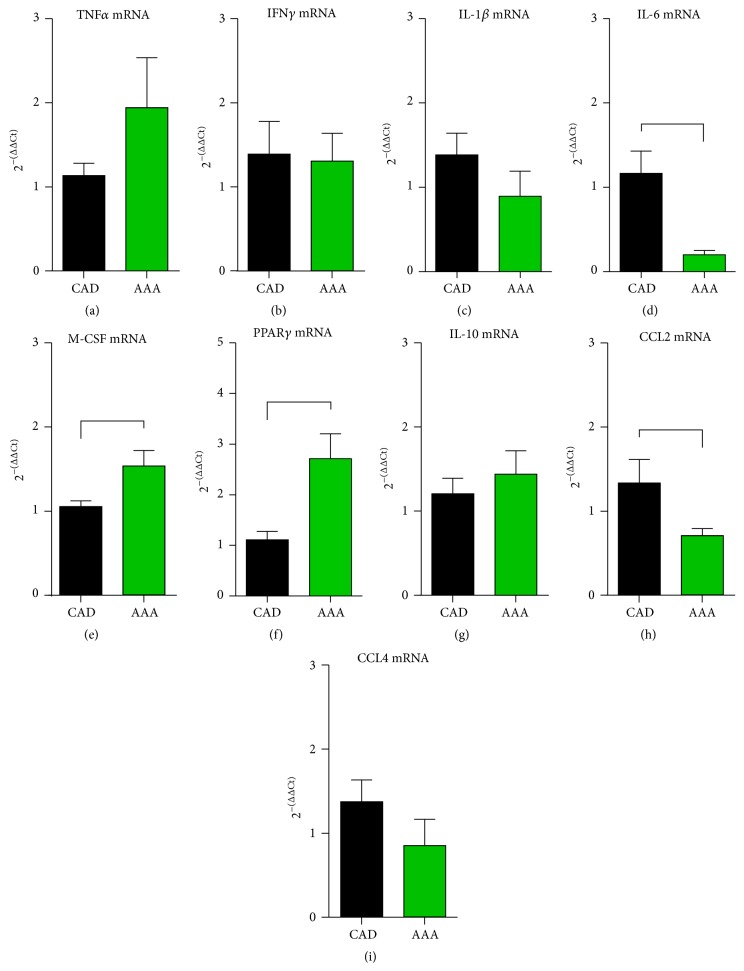
mRNA expression of cytokines and chemokines in aortic wall. The mRNA expression of proinflammatory cytokines (a) TNF*α*, (b) IFN*γ*, and (c) IL-1*β* was comparable between ascending aortas from aneurysms (AAA) and patients with coronary artery disease (CAD). The mRNA expression of (d) IL-6 was lower, while (e) macrophage colony-stimulating factor (M-CSF) and (f) peroxisome proliferator-activated receptor (PPAR)*γ* were significantly higher in AAA than in CAD aortic tissue. The expression of (g) the anti-inflammatory cytokine IL-10 was comparable between the groups. (h) A significantly lower expression of potent mononuclear chemoattractants CCL2 and (i) nonsignificantly lower level of related chemokine CCL4 were found in AAA samples. *n* = 19 in the AAA group and *n* = 30 in the CAD RT-qPCR group. mRNA expression in RT-qPCR is related to controls and GAPDH using comparative ΔΔCt method. Bracket indicates *p* < 0.05.

In the next step, we measured the molecular expression of inflammatory mediators associated with this cellular infiltration pattern. Interestingly, we found no significant difference in mRNA expression of proinflammatory cytokines TNF*α*, IFN*γ*, and IL-1*β* (Figures 3(a)–3(c)), or anti-inflammatory cytokine IL-10 (Figure 3(g)). We measured a significantly lower expression of the cytokine IL-6 (Figure 3(d)) and significantly higher expression of macrophage colony-stimulating factor (M-CSF) (Figure 3(e)) and peroxisome proliferator-activated receptor (PPAR)*γ* (Figure 3(f)) in aneurysms than in controls. In addition, we measured expression of potent mononuclear chemoattractants and found that chemokine CCL2 had significantly lower expression in aneurysms than in controls, while the chemokine CCL4 showed only a tendency to a lower expression in aneurysms (Figures 3(h), 3(i)). We also measured mRNA expression of reactive oxygen scavengers heme oxygenase 1 and glutathione peroxidase 1 and found no difference between the two groups (data not shown). Therefore, the persistent cellular infiltration of the adventitia of aneurysms was associated with an increased M-CSF and PPAR*γ* expression, while the aorta of patients with coronary artery disease showed higher level of chemokine CCL2 and cytokine IL-6.

### 3.3. Collagen and Markers of Remodeling in Aortic Aneurysm

**Figure 4:**
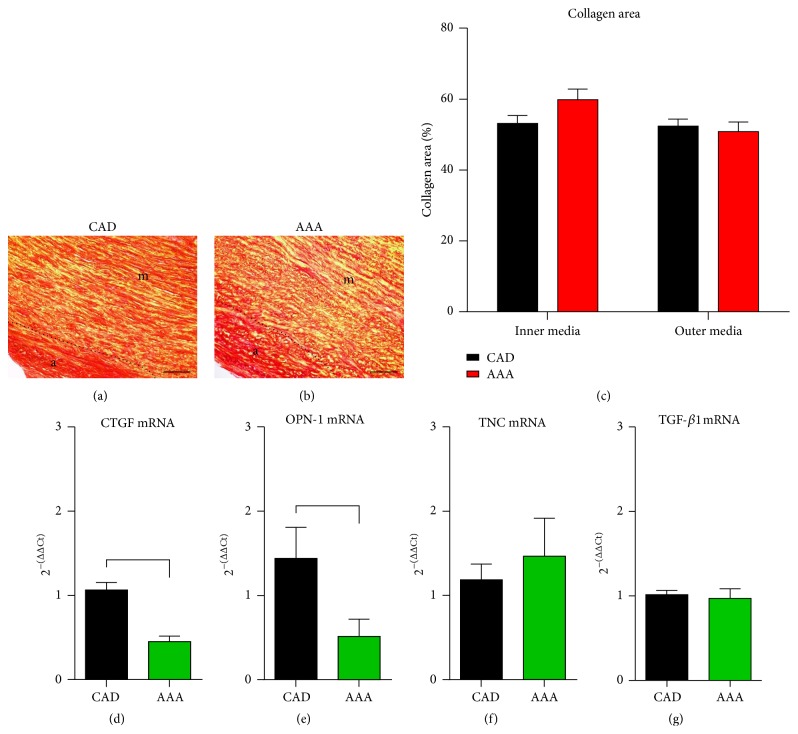
Collagen area and expression of remodeling related factors in aortic aneurysm. Representative images of picrosirius red staining show comparable collagen-deposition pattern in media (m) and adventitia (a) of (a) aorta from patient with coronary artery disease (CAD) and (b) aneurysm of the ascending aorta (AAA). (c) The planimetric evaluation of the collagen-stained area in the inner and outer media showed comparable results between both groups. The mRNA expression of (d) the collagen-related connective tissue growth factor (CTGF) and (e) extracellular matrix remodeling related factor osteopontin-1 is significantly lower in AAA when compared to the CAD aortic samples. (f) The mRNA expressions of early remodeling factor tenascin C and (g) transforming growth factor- (TGF-) *β*1 are comparable between the groups. *n* = 19 in the AAA group, *n* = 24 in the CAD histology, and *n* = 30 in the CAD RT-qPCR group. mRNA expression in RT-qPCR is related to controls and GAPDH using comparative ΔΔCt method. Bracket indicates *p* < 0.05.

**Figure 5:**
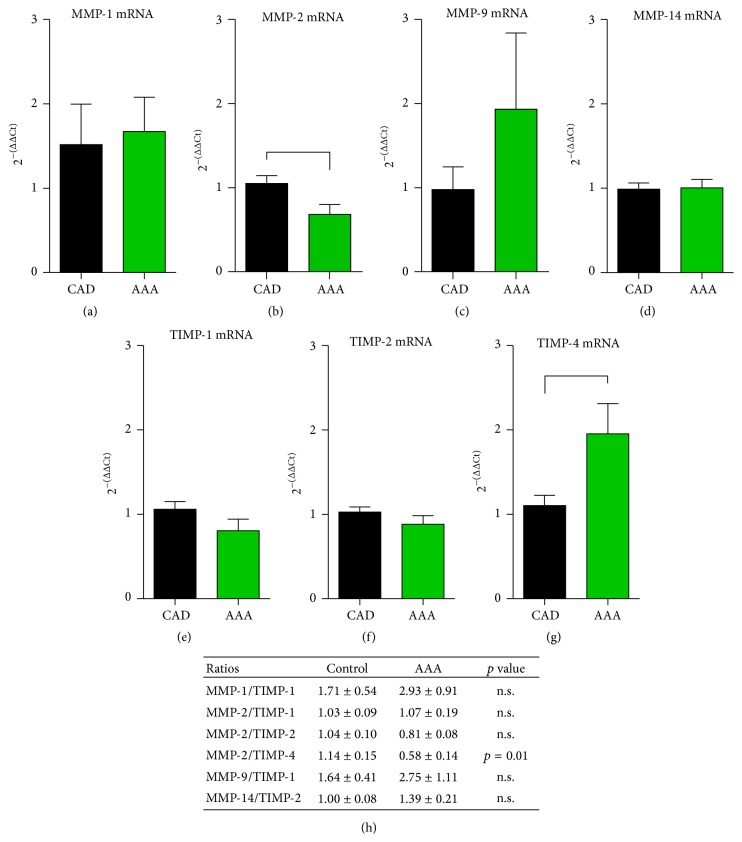
Expression of matrix metalloproteinases and their tissue inhibitors in aortic aneurysm. The mRNA expression of (a) matrix metalloproteinase- (MMP-) 1, (b) MMP-2, (c) MMP-9, and (d) MMP-14 was not significantly different in aorta from patients with aneurysm of the ascending aorta (AAA) compared to coronary artery disease (CAD) except for MMP-2. The mRNA expression of (e) tissue inhibitor of matrix metalloproteinase- (TIMP-) 1, (f) TIMP-2, and (g) TIMP-4 was not significantly different in aorta from patients with AAA compared to CAD except for TIMP-4. (h) Ratios of different MMP/TIMP showed only a significant difference for MMP-2/TIMP-4 between AAA and CAD group. *n* = 19 in the AAA group and *n* = 30 in the CAD RT-qPCR group. mRNA expression in RT-qPCR is related to controls and GAPDH using comparative ΔΔCt method. Bracket indicates *p* < 0.05.

Several experimental and clinical studies showed association between endocannabinoids and tissue remodeling [15, 17], and we therefore compared collagen and related remodeling markers between the aneurysms and controls. Collagen was visualised using picrosirius red staining in the aortic wall and its planimetric evaluation revealed a comparable collagen-stained area between both groups (Figures 4(a)–4(c)). We also differentiated between the inner and the outer tunica media, since the thinner aneurysmatic wall may have less collagen, but we found no difference between the two layers in both groups. Next, we measured mRNA expression of related mediators and found a significantly lower level of connective tissue growth factor (CTGF) and osteopontin-1 in aneurysms (Figures 4(d), 4(e)). At the same time, the expression of early remodeling marker tenascin C was comparable between the groups (Figure 4(f)). We also measured the mRNA expression of transforming growth factor- (TGF-) *β*1 and found no difference between the groups (Figure 4(g)). Since tissue remodeling is always associated with induction of matrix metalloproteinases (MMPs) and their tissue inhibitors (TIMPs), we measured the level of MMP-1, MMP-2, MMP-9, and MMP-14 (Figures 5(a)–5(d)), as well as TIMP-1, TIMP-2, and TIMP-4 (Figures 5(e)–5(g)). We found only a significantly lower mRNA expression of MMP-2 in aneurysms, whereas higher expression of MMP-9 in aneurysms was not significant between the groups as well as the expression of MMP-1 and MMP-14. At the same time, we found no difference in expression of TIMP-1 and TIMP-2. The significantly higher expression of TIMP-4 seems to be responsible for the lower MMP-2 expression and this is also reflected in their significantly increased MMP-2/TIMP-4 ratio (Figure 5(h)). Other MMP/TIMP ratios were not significantly different. These findings indicate a finely regulated low-level extracellular matrix remodeling being driven predominantly by MMP-2/TIMP-1 interaction in aortic aneurysms, when compared to controls.

## 4. Discussion

Several studies investigated mechanisms in pathogenesis of human aortic aneurysm. Beside the genetically well-described diseases [3, 4], there are a growing number of studies reporting molecular mediators and cellular interactions in the aortic aneurysmatic wall. Inflammation has been postulated as a major factor during development of abdominal aortic aneurysms [7] and has not yet been reported in aneurysms of the ascending aorta. Since regulation of inflammatory response and tissue remodeling are the major effects mediated by the endocannabinoid system [14, 15], we investigated it in aneurysms of ascending aorta.

With respect to the published data, we here provide novel evidence for the activation of the endocannabinoid system in human aortic aneurysms. The higher mRNA levels of the four receptors (CB1, CB2, TRPV1, and GRP55) show together with differential levels of their ligands in the tissue an activated endocannabinoid system in human aneurysms. The lower tissue amount of arachidonic acid, a degradation product of both endocannabinoids, also supports this assumption, indicating prolonged demand for endocannabinoids in aneurysmatic tissue. Since the published data suggest a regulatory role of AEA in inflammatory response, the lower AEA tissue amount could be associated with lower expression of IL-6 and CCL2. Still, our data also show higher M-CSF and PPAR*γ* expression, and in the lack of experimental evidence we can only speculate whether this finding is linked to the increased 2-AG tissue amount. To our knowledge, a distinct association of the two known endocannabinoids with specific mediators of inflammatory response has not yet been dissected. The endocannabinoid-mediated fine-tuning of inflammatory reaction involves several mediators acting on different cell types [14]. Our data on the newly recruited leukocytes show no difference between aneurysms and controls but reveal a constant low-level infiltration of them into the adventitia of the aortic wall. This action of inflammatory cells underlines their role in homeostatic regulation of the aortic wall tissue, representing not only inflammation, but also tissue remodeling or regeneration. While previous studies reported mostly on the CD68-positive mature macrophages or T-cells [19], our study also provides evidence for an increased infiltration of the aortic wall by CD11b-positive mononuclear cells. In the context of the endocannabinoid system, the mononuclear cell infiltration is important because of a CB2 receptor-dependent differentiation of M2a subpopulation of macrophages acting at the junction between inflammation and remodeling, as we recently described [17]. In the same study, we also showed a CB2 receptor-dependent regulation of potent inflammatory mediator for mononuclear cells M-CSF in murine heart* in vivo*. Our present study shows a significantly higher level of M-CSF and PPAR*γ* associated with a lower level of IL-6 and no significant difference in other cytokines. Still, we found a significantly lower expression of potent chemotactic mediator chemokine CCL2, which may reflect a negative feedback regulation of leukocyte infiltration in order to keep the inflammation at a very low level. This finding is very interesting since experimental studies have suggested beneficial effects of chemokine inhibition on formation of aneurysms [8], but future studies specifically targeting chemokines should clarify their contribution to this pathology. Another interesting aspect regarding production of reactive oxygen species has also been associated with human aortic aneurysms [10]. Even though we did not measure reactive oxygen species production, we found no difference in the expression of their scavenger enzymes heme oxygenase and glutathione peroxidase. So far, all of these facts indicate a very finely tuned inflammatory process in human aortic aneurysms and therefore the recently postulated prognostic value for phosphorylation of c-kit and downstream targets in aneurysm formation fits well in this concept [20]. In this context, recent studies reported that the inhibition of cytokine IL-1*β* attenuates experimental development of aortic aneurysm [21], as well as activation of TGF-*β* signalling [22]. Future studies should address the exact characterization and cellular imaging of the inflammatory cells, that is, T-cells, macrophage subpopulations, and so forth, in order to better understand their specific contribution during development of human aneurysms.

The homeostasis of vascular tissue is associated with constant remodeling in order to preserve the normal function of the blood vessels. Tissue remodeling involves several mediators acting in extracellular matrix and specific cells, for example, smooth muscle cells or fibroblasts. As a result, in aneurysms, the thickness of the vessel wall changes and this may lead to dissection or rupture. The nature of acquisition of aortic buttons in bypass surgery did not allow measurement of the exact aortic wall thickness. We therefore concentrated on matrix components and investigated the collagen-stained area in the tunica media of the aorta. Induction of CTGF, a major collagen-deposition related factor, was reported in vascular smooth muscle cells after oxidative burst in thoracic aneurysms* ex vivo* [10]. A recent study reported higher level of CTGF, osteopontin-1, and collagen in human thoracic aneurysms when compared to CABG controls without significant difference in incidence of hypertension or diabetes [23]. Their results are in contrast to our data, probably due to the differences in the control group. Our controls have a higher incidence of hypertension and diabetes, which is expected in patients having CAD, as well as WBC count, thus supporting the inflammatory nature of the underlying atherosclerosis. Despite this difference, our aneurysm samples show a higher level of M-CSF, PPAR*γ*, and mononuclear cells than in controls, thereby indicating a specific modulation of vascular remodeling. Even though we report no difference in collagen-stained area between the two groups, we measured significantly lower expression of the CTGF, indicating a lower turnover of the collagen in the aneurysms. Our findings on CTGF are further supported by the lower expression of osteopontin-1, which has been associated with general deposition of matricellular components and tissue remodeling. Expression of tenascin C, an early remodeling marker, is comparable between the two groups and this additionally indicates absence of novel extracellular matrix deposition, but rather a low grade of matrix turnover. Our previous experimental work showed a CB2 receptor-mediated regulation of smooth muscle cell function, where myofibroblasts were not able to induce tenascin C, MMP-9, and TIMP-1 under hypoxic cell culture conditions [17]. In the same study, we observed an increased MMP-9/TIMP-1 ratio and lower myofibroblast density in CB2 receptor-deficient mice in our* in vivo* model of myocardial ischemia. These findings strongly indicate an important role for endocannabinoid system in function of smooth muscle cells and extracellular matrix remodeling. Previous studies reported activation of MMP-2 and MMP-9 being associated with formation of aortic aneurysms [11, 12]. While some experimental data are associated with dissection of the abdominal aorta [24], other studies provide evidence for structural differences between the thoracic and abdominal aorta which in addition to different biophysical stimulus makes them not well comparable [25]. A recent study showed attenuation of experimental aortic aneurysm formation in mice after use of unsaturated fatty acid causing reduced expression of MMP-2 and MMP-9 [26]. Our data describe a significantly lower expression of MMP-2 and higher expression of TIMP-4 in aneurysms with no significant difference in other MMPs or TIMPs. Since TIMP-4 is an important counter actor for MMP-2 in achieving a balanced matrix turnover, we assume that the observed difference in MMP-2/TIMP-4 ratio reflects this balance in our aneurysm patients. Our MMP-2 findings are in contrast to some of the published data [11, 12] and we assume this difference to be based on practically unknown stage of disease development in our patients and other human studies, abdominal versus thoracic aortic tissue, differences between animal and human tissue, or even different extent of inflammation in the aorta in experimental settings including dissection. Future studies are needed to clarify these differences.

This study has a relatively small patient number and in the lack of an experimental model it offers only descriptive data regarding the underlying mechanisms. Therefore, examination of suitable genetically or pharmacologically engineered animal models without dissection should be performed in future studies. Endocannabinoids have been associated in experimental studies with arterial pressure regulation, which by now did not yield a specific pharmacological target [27], whereas its association in metabolic homeostasis and diabetes development offers some promising cues [28]. Despite effects on hypertension or diabetes and inability to clearly delineate them from other pathologies, the short action of endocannabinoids due to their fast degradation makes us confident to claim the observed results as representative for the endocannabinoid level in the aortic wall. Our separate analysis of each CAD subgroup showed only a few nonessential differences between the subgroups, and this strengthened the basis for our data interpretation. We have chosen patients undergoing coronary bypass surgery as controls, due to the availability of vital tissue needed for lipid measurements and because patients with aortic aneurysm and age >40 years frequently present with atherosclerotic lesions. Still, our data provides approximation in the extent of atherosclerotic changes in the aorta of control patients when compared to the aneurysms, where both groups showed signs of atherosclerosis in more than 50% of patients. Obviously, aorta from normal healthy subjects would be a “proper” control, but since it is only available postmortem it is not usable for mRNA or endocannabinoid extraction. We could only investigate the tissue at the time point of surgery and therefore one cannot make any assumptions regarding the dynamic changes in tissue remodeling and formation of aortic aneurysms. Therefore, novel imaging techniques and molecular markers in the blood stream are needed in order to better understand the temporal resolution of this process. The translation of the published data into clinical setting has not been achieved yet and therefore the clinical morbidity and outcome of the modern therapy options remain unchanged [29].

## 5. Conclusions

Our study provides evidence for differential regulation and activation of the endocannabinoid system in human aortic aneurysms. This was associated with a low level of inflammatory cell infiltration and induction of M-CSF and PPAR*γ* accompanied by downregulation of cytokine IL-6 and chemokine CCL2. At the same time, vascular remodeling of the aneurysmatic aortic wall was characterized by a lower expression of CTGF, osteopontin-1, and MMP-2, together with increased TIMP-4, all suggesting a possible low collagen and matrix turnover. These findings provide a basis for future pharmacological and experimental studies and may contribute to the development of novel therapeutic targets.

## Figures and Tables

**Table 1:** Preoperative patient data.

	CAD	CAD subgroups			AAA
	Total	Histology	PCR	LC-MRM	
	*n* = 73	*n* = 24	*n* = 30	*n* = 25	*n* = 19
Age (yrs)	69.29 ± 1.19	71.42 ± 1.77	66.67 ± 2.05	70.24 ± 1.86	65.42 ± 2.99
	*p* = 0.29	*p* = 0.15	*p* = 0.82	*p* = 0.29	

Female gender (%)	21.92	29.17	20.00	24.00	31.58
	(16/73)	(7/24)	(6/30)	(5/25)	(6/19)
	*p* = 0.38	*p* = 1.0	*p* = 0.50	*p* = 0.74	

BMI	27.24 ± 0.72	27.69 ± 3.87	27.96 ± 4.29	27.08 ± 0.81	27.60 ± 0.46
	*p* = 0.06	^*∗*^ *p* = 0.03	^*∗*^ *p* = 0.02	*p* = 0.16	

Coronary artery disease (%)	100.00	100.00	100.00	100.00	15.79
	(73/73)	(24/24)	(30/30)	(25/25)	(3/19)
	*p* < 0.01	*p* < 0.01	*p* < 0.01	*p* < 0.01	

Mean aneurysm diameter (cm)					5.40 ± 0.14

AAA (%)	0.00	0.00	0.00	0.00	100.00
	(0/73)	(0/24)	(0/30)	(0/25)	(19/19)
	*p* < 0.01	*p* < 0.01	*p* < 0.01	*p* < 0.01	

Aortic valve regurgitation (%)	9.59	8.34	6.67	12.00	57.90
	(7/73)	(2/24)	(2/30)	(3/25)	(11/19)
	^*∗*^ *p* < 0.01	^*∗*^ *p* < 0.01	^*∗*^ *p* < 0.01	^*∗*^ *p* < 0.01	

Aortic valve stenosis (%)	13.70	16.67	16.67	4.00	21.05
	(10/73)	(4/24)	(5/30)	(1/25)	(4/19)
	*p* = 0.48	*p* = 1.0	*p* = 0.72	*p* = 0.15	

Bicuspid aortic valve	1.37	0.00	1.37	0.00	21.05
	(1/73)	(0/24)	(1/30)	(0/25)	(4/19)
	^*∗*^ *p* < 0.01	^*∗*^ *p* = 0.03	*p* = 0.07	^*∗*^ *p* = 0.03	

Ejection fraction (%)	55.32 ± 1.35	58.92 ± 2.48	54.17 ± 2.13	52.96 ± 1.80	55.89 ± 2.43
	*p* = 0.84	*p* = 0.4	*p* = 0.6	*p* = 0.33	

Atrial fibrillation (%)	19.18	29.17	13.33	12.00	15.79
	(14/73)	(7/24)	(4/30)	(3/25)	(3/19)
	*p* = 1.0	*p* = 0.47	*p* = 1.0	*p* = 1.0	

Hypertension (%)	93.15	83.33	96.67	100	68.42
	(68/73)	(20/24)	(29/30)	(25/25)	(13/19)
	^*∗*^ *p* < 0.01	*p* = 0.30	^*∗*^ *p* = 0.01	^*∗*^ *p* < 0.01	

Stroke (%)	20.55	12.50	23.33	20.00	5.26
	(15/73)	(3/24)	(7/30)	(5/20)	(1/19)
	*p* = 0.18	*p* = 0.62	*p* = 0.13	*p* = 0.21	

Diabetes (%)	41.10	16.67	50.00	44.00	5.26
	(30/73)	(4/24)	(15/30)	(11/25)	(1/19)
	^*∗*^ *p* < 0.01	*p* = 0.36	^*∗*^ *p* < 0.01	^*∗*^ *p* = 0.01	

COPD (%)	5.48	0.00	6.67	8.00	5.26
	(4/73)	(0/24)	(2/30)	(2/25)	(1/19)
	*p* = 1.0	*p* = 0.44	*p* = 1.0	*p* = 1.0	

Renal dysfunction (%)	16.44	8.33	13.33	24.00	5.26
	(12/73)	(2/24)	(4/30)	(6/25)	(1/19)
	*p* = 0.29	*p* = 1.0	*p* = 0.64	*p* = 0.12	

Creatinine (mg/dL)	1.05 ± 0.04	0.97 ± 0.05	1.05 ± 0.05	1.15 ± 0.08	1.02 ± 0.04
	*p* = 0.06	*p* = 0.37	*p* = 0.74	*p* = 0.17	

Troponin (ng/dL)	0.39 ± 0.17	0.18 ± 0.097	0.65 ± 0.44	0.23 ± 0.12	0.03 ± 0.01
	*p* = 0.09	*p* = 0.13	*p* = 0.08	*p* = 0.78	

Hemoglobin (g/dL)	13.77 ± 0.18	13.87 ± 0.3	13.63 ± 0.3	13.50 ± 0.35	14.27 ± 0.27
	*p* = 0.19	*p* = 0.33	*p* = 0.14	*p* = 0.11	

WBC count (G/L)	7.8 ± 0.26	8.09 ± 0.4	7.7 ± 0.41	7.43 ± 0.49	6.45 ± 0.3
	^*∗*^ *p* = 0.01	^*∗*^ *p* < 0.01	^*∗*^ *p* = 0.03	*p* = 0.11	

CRP (mg/L)	6.60 ± 1.87	4.19 ± 0.87	4.18 ± 1.01	6.04 ± 2.09	4.80 ± 2.12
	*p* = 0.14	*p* = 0.28	*p* = 0.29	*p* = 0.46	

Renal dysfunction was defined with serum creatinine above the normal range of 0.6–1.3 mg/dL. Normal range of laboratory parameters are troponin <0.05 ng/mL, haemoglobin 12.0–15.4 g/dL, WBC count 3.9–10.2 G/L, and CRP < 3 mg/L. CAD: coronary artery disease, AAA: aneurysm of ascending aorta, BMI: body mass index, COPD: chronic obstructive pulmonary disease, WBC: white blood cell, and CRP: C-reactive protein. Data presented as mean ± SEM. ^*∗*^ *p* < 0.05 versus AAA.

**Table 2:** Surgery and postoperative patient data.

	CAD	CAD subgroups			AAA
	Total	Histology	PCR	LC-MRM	
	*n* = 73	*n* = 24	*n* = 30	*n* = 25	*N* = 19
Aortic prosthesis (%)	0.00	0.00	0.00	0.00	100.00
	(0/73)	(0/24)	(0/30)	(0/25)	
	^*∗*^ *p* = 0.01	^*∗*^ *p* = 0.01	^*∗*^ *p* = 0.01	^*∗*^ *p* = 0.01	

Aortic valve replacement (%)	15.07	16.67	13.33	12	42.10
	(11/73)	(4/24)	(4/30)	(3/25)	(8/19)
	^*∗*^ *p* = 0.02	*p* = 0.09	^*∗*^ *p* = 0.04	^*∗*^ *p* = 0.04	

Aortic valve repair (%)	0.00	0.00	0.00	0.00	15.79
	(0/73)	(0/24)	(0/30)	(0/25)	(3/19)
	^*∗*^ *p* = 0.01	^*∗*^ *p* = 0.01	^*∗*^ *p* = 0.01	^*∗*^ *p* = 0.01	

CABG surgery (%)	100	100	100	100	10.50
	(73/73)	(24/24)	(30/30)	(25/25)	(2/19)
	^*∗*^ *p* < 0.01	^*∗*^ *p* < 0.01	^*∗*^ *p* < 0.01	^*∗*^ *p* < 0.01	

Perioperative stroke (%)	0.00	0.00	0.00	0.00	0.00
	(0/73)	(0/24)	(0/30)	(0/25)	(0/19)
	*p* = 1.0	*p* = 1.0	*p* = 1.0	*p* = 1.0	

Hemoglobin (g/dL)	10.48 ± 0.16	10.64 ± 0.29	10.33 ± 0.25	10.59 ± 0.24	10.84 ± 0.31
	*p* = 0.3	*p* = 0.64	*p* = 0.21	*p* = 0.52	

Creatinine (mg/dL)	1.15 ± 0.04	1.02 ± 0.07	1.17 ± 0.08	1.21 ± 0.09	1.01 ± 0.07
	*p* = 0.14	*p* = 0.27	*p* = 0.17	*p* = 0.11	

Troponin (ng/dL)	0.68 ± 0.22	0.43 ± 1.11	0.96 ± 0.47	0.73 ± 0.44	1.09 ± 0.34
	^*∗*^ *p* = 0.02	^*∗*^ *p* = 0.02	*p* = 0.21	*p* = 0.07	

WBC count (G/L)	9.64 ± 0.30	10.26 ± 0.56	9.44 ± 0.36	9.34 ± 0.61	9.47 ± 0.87
	*p* = 0.82	*p* = 0.44	*p* = 0.97	*p* = 0.89	

CRP (mg/L)	70.84 ± 6.00	70.3 ± 11.7	68.00 ± 8.33	69.59 ± 9.53	80.53 ± 9.36
	*p* = 0.43	*p* = 0.51	*p* = 0.33	*p* = 0.43	

Surgery data and laboratory data obtained 72 h postoperatively. Normal range of laboratory parameters are creatinine 0.6–1.3 mg/dL, troponin <0.05 ng/mL, haemoglobin 12.0–15.4 g/dL, WBC count 3.9–10.2 G/L, and CRP < 3 mg/L. CAD: coronary artery disease, AAA: aneurysm of ascending aorta, CABG: coronary artery bypass grafting, WBC: white blood cell, and CRP: C-reactive protein. Data presented as mean ± SEM. ^*∗*^ *p* < 0.05 versus AAA.
